# An Example-Based Super-Resolution Algorithm for Selfie Images

**DOI:** 10.1155/2016/8306342

**Published:** 2016-03-15

**Authors:** Jino Hans William, N. Venkateswaran, Srinath Narayanan, Sandeep Ramachandran

**Affiliations:** Department of ECE, SSN College of Engineering, Chennai, Tamil Nadu 603 110, India

## Abstract

A selfie is typically a self-portrait captured using the front camera of a smartphone. Most state-of-the-art smartphones are equipped with a high-resolution (HR) rear camera and a low-resolution (LR) front camera. As selfies are captured by front camera with limited pixel resolution, the fine details in it are explicitly missed. This paper aims to improve the resolution of selfies by exploiting the fine details in HR images captured by rear camera using an example-based super-resolution (SR) algorithm. HR images captured by rear camera carry significant fine details and are used as an exemplar to train an optimal matrix-value regression (MVR) operator. The MVR operator serves as an image-pair priori which learns the correspondence between the LR-HR patch-pairs and is effectively used to super-resolve LR selfie images. The proposed MVR algorithm avoids vectorization of image patch-pairs and preserves image-level information during both learning and recovering process. The proposed algorithm is evaluated for its efficiency and effectiveness both qualitatively and quantitatively with other state-of-the-art SR algorithms. The results validate that the proposed algorithm is efficient as it requires less than 3 seconds to super-resolve LR selfie and is effective as it preserves sharp details without introducing any counterfeit fine details.

## 1. Introduction

With the advent of smartphones having sophisticated camera technologies and integrated online social networking services, selfies gain popularity among social media users. Selfie is typically a photograph that one has taken of oneself, using the front camera of a smartphone. Most conventional smartphones have two cameras, a primary rear camera and a secondary front camera. As the front camera is mainly intended for video conference, it has limited pixel resolution compared with rear camera. For instance, Apple's iPhone 6 has a 1.2-megapixel (MP) front camera which is very much limited compared with primary 8 MP rear camera in terms of pixel resolution. Though the front camera is designed for video conference, it is often used by users to capture selfies. Selfies are low-resolution (LR) images, as the fine details in it are explicitly missed due to hardware limitation of the front camera. Despite the fact that selfies are self-portraits which essentially comprise facial information of the user, it is equally important to proclaim the importance of the background information in it. The vital background information can be an interesting scene, astounding location, or a group of friends. Selfies are widely shared via social media; hence the volume of such images is burgeoning and there is a need to improve the quality of these images.

Super-resolution (SR) algorithm [1] aims to generate high-resolution (HR) image from single or ensemble of LR images. Example-based SR algorithms [2–4] enhance the resolution of LR image by learning the high frequency (HF) details from LR-HR training examples. The priori which defines the relation between the LR and HR images could be learned from the training image-pairs. The learned image-pair priori [5] can be used to generate HR image from the observed LR image. Conventional example-based SR algorithms can be characterized into two categories with respect to the way image-pair priori is learned from the training set, namely, the implicit- and explicit-priori based methods. The implicit-priori based algorithms [6–8] represent the priori directly from the training image-pairs. Most of the traditional *K*-nearest neighbor algorithms [6, 9] are implicit and are computationally expensive to search the *K*-nearest neighbors to estimate the HR image. The explicit-priori based algorithms either use a dictionary [10–12] or a regression function [13, 14] to map the correspondence between the LR-HR image-pairs. Dictionary based algorithms [15, 16] represent the priori between LR and HR image-pairs by a LR-HR dictionary pair. In regression based approaches, the regression function which maps the LR and HR image-pairs can be mapped by either a supervised [13] or semisupervised [17] learning process. The time required to train explicit image-pair priori is generally high. Therefore, conventional example-based SR algorithms are not suitable for super-resolving selfies.

The main challenge in super-resolving LR selfie is to learn the image-pair priori which maps the LR to HR image-level correspondence with minimum computational complexity. As HR images captured by the rear camera preserve fine details, it can be used to learn a priori to super-resolve selfies. Most of the conventional example-based SR algorithms are implemented by vectorizing the training image-pairs [9, 15]. By vectorizing, the image-level information between image-pairs is lost due to structural disparity. Hence the vector-based priori which relates the LR-HR image-pairs is not effective [18]. To overcome this difficulty, a novel matrix-based priori is proposed by Tang and Yuan [18] to model the image-pair priori. However, the matrix-based priori is derived based on the assumption that most of the image patches extracted from natural training images are full rank [18]. Though this assumption is valid for natural images, patches extracted from real-life images with facial information and smooth textures are intuitively rank deficient.

This paper endeavors to improve the spatial resolution of selfies by efficiently learning an optimal matrix-value regression (MVR) operator from LR-HR image patch-pairs extracted from training samples captured by rear camera of the smartphone. The training image patch-pairs are factorized by singular value decomposition (SVD) to accommodate rank deficient patch-pairs in the learning process. The MVR operator explicitly models the correspondence between the LR- and HR-training image patches to super-resolve the LR selfies. As the proposed MVR algorithm avoids vectorization, it preserves the structural similarity of training image patches and enjoys image-level information within them. The computational cost of the proposed algorithm is greatly reduced by optimally selecting a larger patch-size in both training and recovering phase as it carries significant image-level information. The main contributions of this paper are as follows:

- *A fast selfie SR algorithm:* LR selfies are super-resolved by a fast example-based algorithm using an optimal MVR operator learned from HR training images captured by rear camera of the smartphone.
- *Effective and efficient MVR operator:* The computational cost to learn the MVR operator is minimum. Also, it faithfully preserves the structural similarity between training image patch-pairs, which makes the MVR operator effective and efficient.

The remainder of the paper is organized as follows. A brief description on image-pair analysis methods is reported in Section 2. In Section 3, the proposed SR methodology for selfie images is explained in detail. In Section 4, experimental evaluations are reported to compare the performance of the proposed method and finally Section 5 concludes the paper.

## 2. Brief Description on Image-Pair Analysis Methods

**Figure 1:**
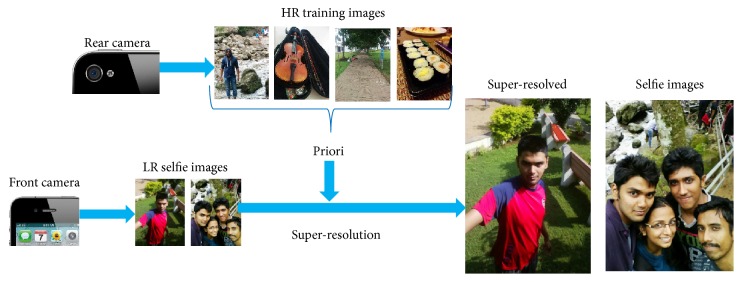
Overview of example-based super-resolution.

Example-based super-resolution algorithms estimate the fine details that are missed in LR images by learning the correspondence between training image-pairs. The process of example-based super-resolution is summarized in Figure 1. Effective image-pair analysis methods are required by example-based SR algorithms to learn an image-pair regression operator, which defines a relation between LR-HR image-pairs. Training image-pair typically consists of a HR image and its corresponding synthetically generated LR image. Well learned image-pair regression operator provides significantly precise correspondence between LR-HR patch-pairs and could be effectively used as a global priori in many inverse image processing tasks [19, 20]. Example-based SR is an ill-posed problem and requires sophisticated image-pair analysis methods [18] to learn the suitable regression operator from training examples.

Image-pair analysis methods are classified as vector-based and matrix-based methods. In vector-based image-pair analysis methods [15, 16], LR-HR image patch-pairs are represented as feature vectors and its correspondence is learned with an explicit vector-based regression operator. Though image patch-pairs are faithfully represented as vectors in vector-based methods, its image-level structural information is lost due to vectorization [21, 22]. Therefore the problem of image-pair analysis is converted to a problem of vector-pair analysis. To avoid structural disparity and preserve image-level information within patch-pairs, a few matrix-based image-pair analysis methods are suggested [18, 23]. In these methods [18, 23], a linear matrix-based regression operator is learned to map the global dependency [18] between LR-HR patch-pairs.

### 2.1. Matrix-Value Regression (MVR) Operator

An image patch-pair denoted as *s* = (*x*, *y*) ∈ *ℝ* ^*p*×*p*^ defines a linear matrix-value regression (MVR) operator *M*  : ∈ *ℝ* ^*p*×*p*^ such that

(1) $$\begin{array}{c} y=M\cdot x. \end{array}$$

If the image patch-pairs are assumed to be full rank matrices, then the MVR operator *M* can be obtained as

(2) $$\begin{array}{c} M=yx^{-1}, \end{array}$$

where *x* ^−1^ refers to the matrix inverse of *x*.

The MVR operator *M* profoundly depends on the full rank condition of its constituent patch-pairs to compute the matrix inverse. For rank deficient matrices, computing inverse is not stable. Hence, in recent matrix-based image-pair analysis methods [18, 23, 24] the patch-pairs are assumed to be full rank matrices. However, the main difference between a selfie image and general image is with respect to its information content. Typical selfie images essentially carry the facial information of the user that contains a foreground with vivid facial features of similar textures and a background with less complex information. However, general images will carry any natural information having more complex structures with random patterns and textures [25]. Image patches extracted from random natural images are intuitively assumed to be full rank [18] due to complex structures in it. Though this assumption is valid (ideally producing 5% rank deficiency) for natural images, image patches extracted from selfie images are intuitively rank deficient. To validate this, an experiment was carried out with 100000 patches extracted from training images and it is observed that approximately 50% of the patches are rank deficient as shown in Table 1. This is attributed to the similar texture details present in the training samples. Furthermore, this percentage increases for larger patch-size as the patch coherence becomes higher. To accommodate rank deficient patch-pairs to represent the image-pair priori, matrix inverse is computed by factorizing the patch-pairs with singular value decomposition.

### 2.2. Similarity Measure via MVR Operator

The linear MVR operator *M* precisely models the correspondence between the image patch-pairs *s*_*i*_ = (*x*, *y*)_*i*=1_^*n*^ ∈ *ℝ* ^*p*×*p*^. Therefore from (1), we get

(3) $$\begin{array}{c} M\cdot x=\left(\;y_{i}x_{i}^{-1}\;\right)x=y_{i}\left(\;x_{i}^{-1}x\;\right)=y. \end{array}$$

If a LR test patch *x* is identical with *i*th patch *x*_*i*_ in the training set, then (*x*_*i*_^−1^ *x*) becomes an identity matrix. The term (*x*_*i*_^−1^ *x*) can be observed as a patch-similarity measure which defines the mutual information between *x* and *x*_*i*_. From (3), the HR estimation *y* of the LR test image *x* can be found effectively using the MVR operator.

### 2.3. Computational Efficiency via MVR Operator

The MVR operator significantly reduces the computational complexity by reducing the number of variables required to represent the operator. As the image patch-pairs are matrices of size *p* × *p*, the image-pair regression operator will be a matrix of size *p* × *p*. Therefore it is required to have *p* ^2^ variables to represent the matrix-based regression operator. Nevertheless, in vector-based approaches, as image patches are column vector of size *p* ^2^ × 1, the regression operator that maps the two vectors should be a matrix of size *p* ^2^ × *p* ^2^ and hence requires *p* ^4^ variables.

## 3. The Proposed Selfie Super-Resolution Methodology

**Figure 2:**
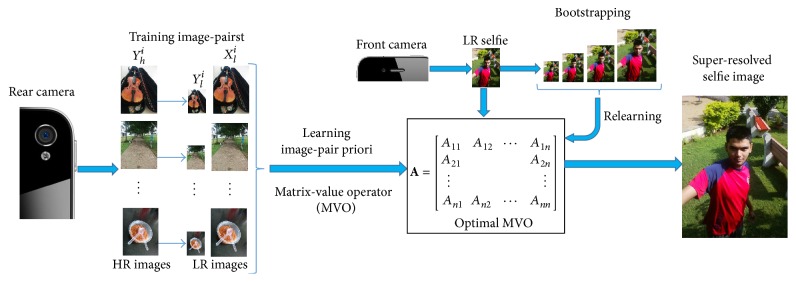
Overview of proposed selfie super-resolution algorithm.

The overview of the proposed selfie SR methodology is illustrated in Figure 2. The example-based selfie SR algorithm consists of a training phase (performed offline), where an optimal MVR operator is learned from a set of image patch-pairs extracted from the training image set and a reconstruction phase performing super-resolution on the test selfie image using the learned matrix-value regression (MVR) operator from the previous phase.

### 3.1. Training Set Construction

The training phase begins by collecting a few HR images {*Y*_*h*_^*i*^ ∈ *ℝ* ^*m*×*n*^}, captured by the rear camera of the smartphone, which are considered as HR examples. Each of these HR images is downscaled by a scale-factor *s*. These downscaled images form the corresponding LR images {*Y*_*l*_^*i*^ ∈ *ℝ* ^(*m*/*s*)×(*n*/*s*)^}. To avoid resolution disparity, the LR images are upscaled to the size of the target HR image by an interpolation operator *Q* : *ℝ* ^(*m*/*s*)×(*n*/*s*)^ → *ℝ* ^*m*×*n*^ and are denoted by {*X*_*l*_^*i*^ ∈ *ℝ* ^*m*×*n*^}. The set of images in *S* = {*Y*_*h*_^*i*^, *X*_*l*_^*i*^} forms the training image-pairs. Let *x* and *y* denote image patches of size (*p* × *p*) extracted from *X*_*l*_ and *Y*_*h*_, respectively. For every image patch *y* extracted from the HR image *Y*_*h*_ centered at its origin (*i*, *j*), there exists a self-similar example patch [25] *x* around its origin (*i*_*s*_, *j*_*s*_) in the LR image *X*_*l*_, where *i*_*s*_ = [*i*/*s* + 0.5] and *j*_*s*_ = [*j*/*s* + 0.5]. The correspondence between *y* and *x* is learned by an optimal MVR operator.

### 3.2. Algorithm to Learn Optimal MVR Operator

Let the training patch-pairs be denoted as *S*_*n*_ = (*x*_*i*_, *y*_*i*_)_*i*=1_^*n*^ ∈ *ℝ* ^*p*×*p*^, where (*x*_*i*_, *y*_*i*_) is low- and high-resolution patch-pairs of size *p* × *p* and *n* is the number of training patch-pairs. Let *M* : *X* ↦ *Y* be a MVR operator mapping the low-resolution image space to the high-resolution image space.

The optimal MVR operator *M* ^*∗*^ is subsequently learned from the training set *S*_*n*_ using the least square regression model given by

(4) $$\begin{array}{c} M^{*}=\underset{M}{\arg\;\min}\overset{n}{\underset{i=1}{\sum }}{\left\|\;y_{i}-Mx_{i}\;\right\|}_{F}^{2}, \end{array}$$

where ‖·‖_*F*_ is the Frobenius norm. Let *F*_*i*_(*M*) be the cost function such that (4) becomes

(5) $$\begin{array}{c} M^{*}=\underset{M}{\arg\;\min}\overset{n}{\underset{i=1}{\sum }}F_{i}\left(\;M\;\right), \end{array}$$

where

(6) FiMyi−MxiF2=yiF2−2yi,MxiF+Mxi,MxiF=yiF2−2yixiT,MF+MxixiT,MF.

To obtain the optimal MVR operator, the target function is given by

(7) FM=∑i=1nFiM=∑i=1nyiF2−2yixiT,MF+MxixiT,MF=∑i=1nyiF2−2∑i=1nyixiT,MF+M∑i=1nxixiT,MF=K0−2K1,MF+MK2,MF,

where *K*_0_ = ∑_*i*=1_^*n*^‖*y*_*i*_‖_*F*_^2^, *K*_1_ = ∑_*i*=1_^*n*^ *y*_*i*_ *x*_*i*_^*T*^, and *K*_2_ = ∑_*i*=1_^*n*^ *x*_*i*_ *x*_*i*_^*T*^ are the auxiliary matrices.

The optimal MVR operator *M* ^*∗*^ can be deduced by imposing condition for minimization on (7); hence

(8) ∂∂MFM=0,∂∂MK0−2K1,MF+MK2,MF=0.

Therefore, the optimal MVR operator is given by

(9) $$\begin{array}{c} M^{*}=K_{1}K_{2}^{-1}. \end{array}$$

The inverse of the auxiliary matrix *K*_2_ is computed by factorizing *K*_2_ with SVD; thus *K*_2_ = *U*Σ*V* ^*T*^, where *U* and *V* are orthogonal matrices and Σ is a diagonal matrix with singular values. Thus

(10) $$\begin{array}{c} M^{*}=K_{1}\left(\;V\Sigma^{-1}U^{T}\;\right). \end{array}$$

The optimal MVR operator *M* ^*∗*^ shown in (10) explicitly represents the image-level correspondence between the low- and high-resolution image patch-pairs. The MVR operator resulting from the training phase is used to reconstruct the fine details from the low-resolution selfie images. The procedure to deduce optimal MVR operator is summarized in Algorithm 1.

### 3.3. Algorithm for SR Reconstruction

In the reconstruction phase, LR selfies captured by the front camera are super-resolved using the MVR operator learned from Algorithm 1. In addition, the MVR operator is adapted to learn from the test selfie itself by a bootstrapping approach [16]. The given test selfie is assumed to be the HR image and the scaled-down version is its LR counterpart. The correspondence between the LR-HR patch-pairs extracted from the bootstrapped image-pairs is used to update the optimal MVR operator. The test selfie *T*_lr_ is interpolated by a factor *s* with an interpolation operator *Q*. Nonoverlapping image patches of size *p* × *p* are extracted from the interpolated test image. This collection of low-resolution patches is represented as *T*_lr_ = {*t*_lr_^*i*^}_*i*=1_^*n*^. Every test LR image patch in set *T*_*s*_ is super-resolved using the optimal MVR operator, such that

(11) $$\begin{array}{c} t_{hr}=M^{*}t_{lr}. \end{array}$$

The super-resolved test image patches are merged to form the super-resolved high-resolution image *T*_hr_. The steps involved in the reconstruction phase are summarized in Algorithm 2.

## 4. Results and Discussions

**Figure 3:**
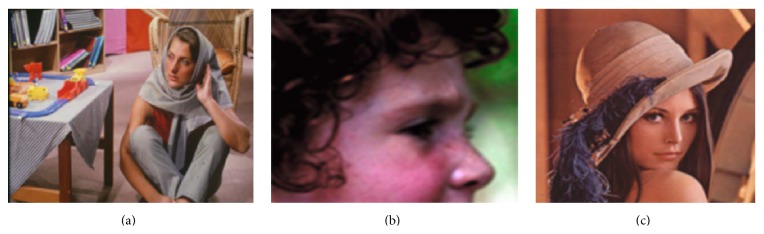
Standard test images.

**Figure 4:**
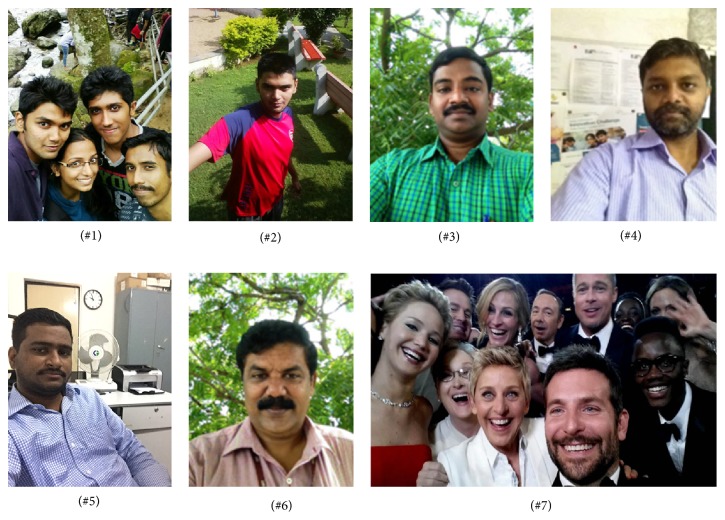
Test selfie images.

The proposed algorithm is evaluated for its effectiveness and efficiency by conducting both qualitative and quantitative experiments on various test images shown in Figures 3 and 4. The test images are super-resolved using state-of-the-art approaches such as Yang et al.'s sparse representation based algorithm [15], Kim et al.'s sparse regression algorithm [9], Dong et al.'s nonlocal autoregressive modeling (NARM) algorithm [26], and He et al.'s Gaussian process regression algorithm [27] and their performance metrics are estimated and compared. Among the algorithms chosen for comparison, Yang et al.'s, Kim et al.'s, and the proposed algorithm are training-based algorithm, whereas Dong et al.'s and He et al.'s algorithm are training-free algorithm. The results of the aforementioned algorithms are obtained using the source codes available at the author's homepage.

### 4.1. Experimental Setup

In the experiments carried out, test images shown in Figures 3 and 4 are used as LR images. Though the algorithm is proposed to super-resolve LR selfie images, few standard test images (shown in Figure 3) such as Barbara, girl, and Lena are used to fairly compare the performance of the proposed algorithm with other state-of-the-art SR algorithms.

**Figure 5:**
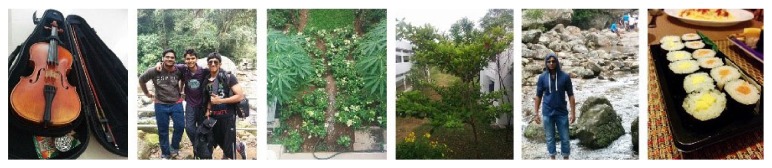
Sample training images.

To evaluate the effectiveness of the proposed algorithm on selfies, various test selfies captured by different smartphones such as iPhone 4s, iPhone 6, and Nexus 5 with diverse specifications are collected.  Figure 4 shows the selfie test images used for comparison, in which images (#1) and (#2) show the selfie test images captured by Nexus 5 with a resolution of 2 MP and (#3) and (#4) depict the selfie test image captured by iPhone 4s with a pixel resolution of 1 MP. Images (#5) and (#6) represent the selfie test image captured by iPhone 6 with a spatial resolution of 1.2 MP and (#7) shows the famous Oscar selfie image (courtesy Google image). The training dataset is generated from a collection of HR images captured by the rear camera of the smartphone offline. The training dataset is limited to 50 HR images with different poses, exposures extracted from the root directory of the smartphone. However, the number of training examples can be extended by adding more examples to the training set. The HR images captured by Nexus 5 have a pixel resolution of 12 MP and iPhone 6 and iPhone 4s have a pixel resolution of 8 MP. Sample training HR images are shown in Figure 5.

The training and testing color images are converted to YCbCr channel and only the luminance channel is considered for super-resolution as it is sensitive to human eye. The LR images are synthetically generated by downsampling the test images shown in Figures 3 and 4 using bicubic interpolator. The downsampled LR images are resized to the size of target HR image and are contiguously blocked into nonoverlapping patches of size 27 × 27. The LR test images are super-resolved by a scale-factor of *s* = 2, 3, and 4. LR-HR training image-pairs are generated with the same scale-factor *s*. All the experiments were carried out using Matlab R2012 on an Intel core i5-2400@2.7 GHz processor with 4 GB RAM.

### 4.2. Experimental Analysis

*Effectiveness*. Qualitative and quantitative evaluation are carried out to assess the effectiveness of the proposed algorithm. Qualitative evaluation of SR methods relies on a few attributes of the reconstructed image such as sharpness, naturalness, and granularity [28]. The sharpness of an image is assessed based on the HF details it preserves. The naturalness of an image is affected by the artifacts present in it. Various artifacts such as ghosting, ringing, jagging, and staircase artifacts generally affect the quality of an image. A visual comparison is made to assess the fidelity of the proposed algorithm qualitatively. The effectiveness of the proposed method is quantitatively evaluated based on a few objective performance metrics such as root mean square error (RMSE), peak signal-to-noise ratio (PSNR), and structural similarity (SSIM) index [29]. A high PSNR score indicates that the scaled-up image is free from distortions and effectively reconstructs the HF details. Similarly, a high SSIM value (typically 1) implies that the scaled-up image has a very similar structure to its ground truth. For fair comparison, the standard test images shown in Figure 3 are super-resolved using the proposed method and are compared with the aforementioned algorithms.  Table 2 summarizes the quantitative comparison of various SR algorithms on test images for 3x magnification.

**Figure 6:**
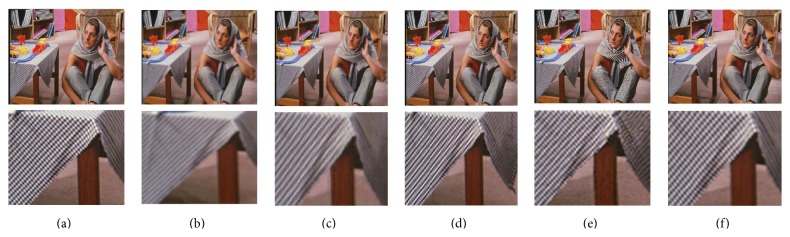
Visual comparison for the Barbara image with other state-of-the-art SR approaches for 2x magnification.

Figure 6 shows the 2x visual comparison for the standard test image Barbara.  Figure 6(a) shows the ground truth and its corresponding scaled-up local image and Figure 6(b) shows the LR image and its corresponding local image. Figures 6(c)–6(e) depict the super-resolved image and its local image by Yang et al.'s algorithm, Kim et al.'s algorithm, and Dong et al.'s algorithm.  Figure 6(f) shows the SR image and its corresponding local image super-resolved by the proposed MVR algorithm. In Figure 6(c), the texture on the table cloth is blurred when compared with ground truth. Though the stripes in the table cloth are sharp in Figure 6(d), it is not the same pattern as in the ground truth as the fine details in the table cloth are not well preserved. Dong et al.'s method reconstructs the texture as in the ground truth; however it introduces ringing and jagging artifacts, as observed in Figure 6(e), and accordingly has low PSNR value. As observed from Figure 6(f), it is evident that the proposed algorithm preserves sharp texture details as in ground truth and is free from artifacts.

**Figure 7:**
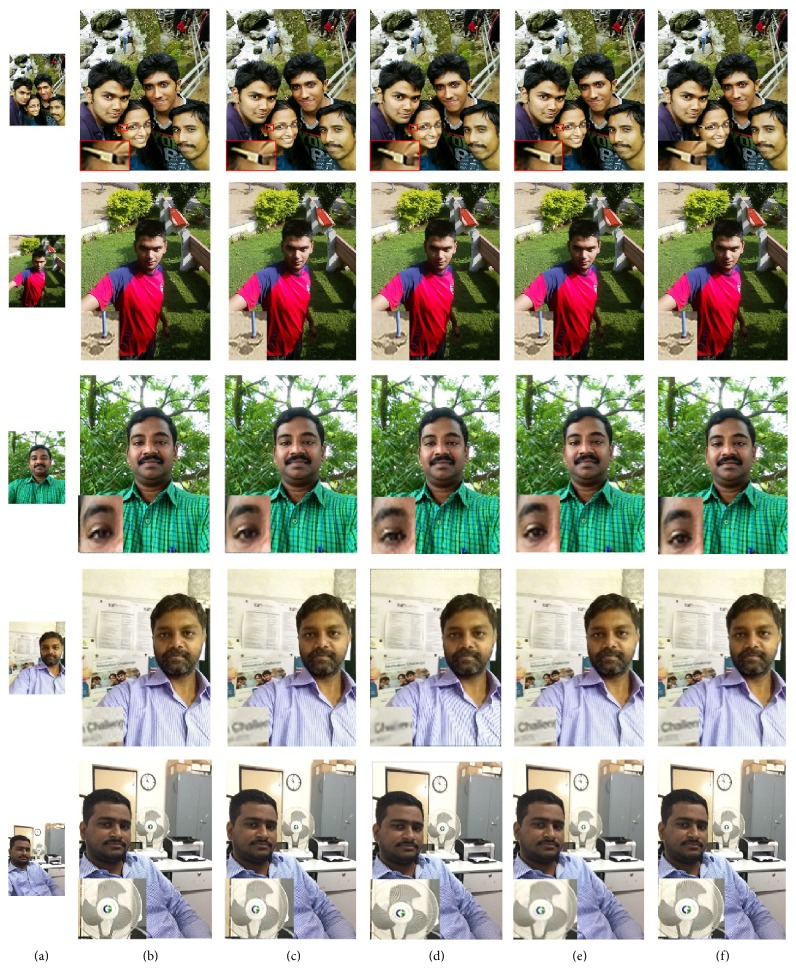
Visual comparison for test selfies with state-of-the-art SR algorithms for 3x magnification: (a) test selfie images, (b)–(e) reconstructed SR images by Yang et al. algorithm, Kim et al. algorithm, Dong et al. algorithm, He et al. algorithm, and proposed algorithm, respectively.

For visual comparison on test selfies, 3x magnification on test selfie images is carried out.  Figure 7 depicts the qualitative visual comparison for five test selfie images.  Figure 7(a) depicts the test LR selfie image. Figures 7(b)–7(e) depict the SR images reconstructed by Yang et al.'s, Kim et al.'s, Dong et al., and He et al.'s algorithm.  Figure 7(f) shows the proposed SR image. The local region of interest (ROI) is highlighted in red boxes and is presented in the bottom left corner of the image. In Yang et al.'s SR based on sparse representation [15] model, two coupled dictionaries are trained simultaneously from random raw image patches. Based on a dictionary pretrained from thousands of natural images, Yang et al.'s method seems to produce natural-looking results. Though Yang's algorithm faithfully reconstructs natural-looking images, it can be observed from Table 2 that the objective measures are not the best among other comparative algorithms. This is because the fine details in the image are not well preserved due to the fact that a universal dictionary used in this method fails to represent complex structures accurately. For instance, the spectacle frame in the ROI of test image (#1) shown in Figure 7(b) looks sharp and natural but for the ROI of test image (#4) in Figure 7(b), the structure of the letters is not preserved. Due to the fact that a natural image priori is used to postprocess the SR image, Kim et al.'s [9] method effectively reproduces more visually appealing images. It preserves minute details (the eyelash in the ROI of test image (#3) in Figure 7(c)) in the reconstructed image. The PSNR and SSIM value for Kim et al.'s method is better than other comparative algorithms, as a postprocessing with an image edge priori is carried out on the reconstructed image. Nevertheless, for images with complicated edges, the edge priori tends to introduce ringing artifacts along the corner of edges, which reduces the PSNR and SSIM value. For example, artifacts can be visualized in the fan rails of ROI of test image (#5) in Figure 7(c). In Dong et al.'s [26] method, overly smooth HF details are recovered as in the ROI of test image (#2) in Figure 7(d). Also, it is prone to introduce artifacts as it can be visualized in the ROI of test image (#3) in Figure 7(d). Due to the artifacts in the reconstructed image, the average PSNR and SSIM value is lesser for Dong et al.'s method. It can be observed that the characters in the ROI of test image (#4) in Figure 7(e) are not faithfully reconstructed by He et al.'s [27] Gaussian process regression method. On the contrary, the proposed method preserves the sharp details and fine textures in most of the images without affecting the naturalness of the image. Also it is observed that the proposed method provides more photorealistic details as it does not introduce any counterfeit fine details. The effectiveness of the proposed algorithm is quantitatively validated from the PSNR and SSIM value observed from Table 2. The proposed method achieves the best PSNR and SSIM value which indicates that the proposed algorithm reconstructs the LR image with minimal distortions and a high SSIM value corroborates the effectiveness of the structural similarity which has been preserved by the proposed matrix-based regression algorithm. The proposed method performs better than other state-of-the-art SR approaches as it avoids vectorization of image patch-pairs during training phase of the MVR operator, which intuitively preserves structural similarity and image-level information within patch-pairs. Also, as the MVR operator is trained with HR images captured by the rear camera of the smartphone it effectively corresponds to the relation between LR-HR patch-pairs, thereby improving the performance of the proposed algorithm. For instance, in the highlighted ROI of test image (#1) shown in Figure 7(f), the fine details in the frame of the spectacle are well preserved. Similarly, the shadow of the pole in the ROI of test image (#2) is very clear. In the ROI of test image (#3) shown in Figure 7(f), very fine details in eye such as eyebrow and eyelash are sharp and the HF details are preserved. Also, it is observed that the structure of letters in the ROI of test image (#4) in Figure 7(f) is preserved when compared with other state-of-the-art approaches.

*Efficiency*. The efficiency of the proposed matrix-based SR algorithm is compared with aforementioned algorithms on a personal computer with Intel core i5-2400@2.7 GHz processor with 4 GB RAM.

The computation time required to train and recover the images is reported in Table 3. Among the training-free algorithms (Dong et al. and He et al. algorithms), the average CPU time taken to recover the SR image by He et al.'s Gaussian process regression algorithm [27] is significantly high as the source code available in the author's homepage is not optimized. The NARM based SR algorithm by Dong et al. [26] takes approximately 3~6 minutes to recover the HR image with a magnification factor of *s* = 3. It is witnessed from Table 3 that the training time required by training-based SR algorithms such as Yang et al. and Kim et al. algorithm is significantly high, as it has to extract training image patches from an extensive dataset to train an universal dictionary. Owing to the fact that image patches are represented as matrices and large patches (typically of size 27 × 27) are used in the proposed MVR algorithm, the computational time is significantly less (<a minute), thereby outperforming other state-of-the-art approaches. The experimental results presented in Table 3 reveal that the proposed MVR algorithm can be efficiently applied to super-resolve LR selfie images with minimum computational expense.

### 4.3. Influence of Patch-Size

**Figure 8:**
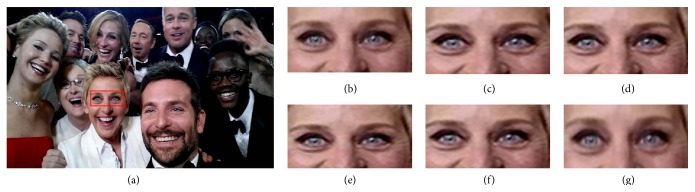
Visual comparison on test selfie (#7) for various patch-sizes: (a) test selfie (#7), (b)–(g) magnified ROI of test selfie (#7) super-resolved with patch-sizes 3 × 3, 11 × 11, 19 × 19, 27 × 27, 35 × 35, and   43 × 43, respectively.

The size of image patch used in training and recovering phase significantly influences the performance of the algorithm. Intuitively, selecting a larger patch-size may produce overly smooth results whereas a small patch tends to produce undesired artifacts in smooth areas of the image. In addition, computational cost of the algorithm is influenced by patch-size. Hence a performance evaluation based on variation in patch-size for the proposed algorithm is carried out and depicted in Figure 5. The magnified ROI highlighted in red box is compared for visual fidelity. In addition, a quantitative analysis based on PSNR for different patch-size is reported in Table 4. The size of training patch is varied from 3 × 3 to 43 × 43 with a step size of 8 pixels. For a small patch-size of 3 × 3 as in Figure 8, the freckles near the eye are relatively blurred and are quantitatively validated in Table 4. The qualitative and quantitative performance of the proposed algorithm increase as the patch-size is increased and are maximum for a patch-size of 27 × 27 as shown in Figure 8 and Table 4, respectively. For instance, it is perceived that the freckles near the eyes are crisper comparatively and hence the eyes look sharper and are natural-looking for a patch-size of 27 × 27 as in Figure 8(e). Due to the fact that the image-level information between patches is preserved by the proposed matrix-based regression algorithm, the performance of the proposed algorithm is better for larger patch-sizes. However, too large patch-size will reduce the performance of the algorithm as it is more complex to utilize the image-level information within them.

### 4.4. Influence of Scale-Factor

**Figure 9:**
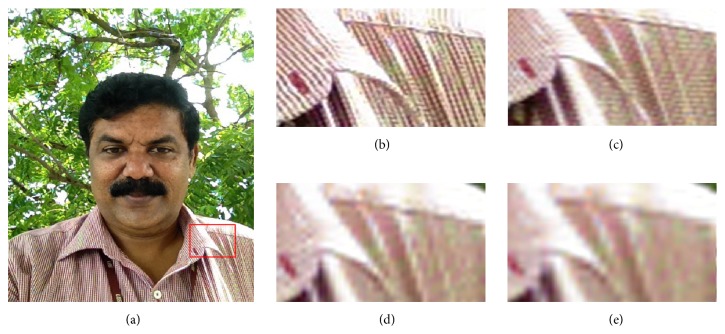
Visual comparison on test selfie (#6) for various scale-factor *s* (a) test selfie (#6), (b)–(e) magnified ROI of test selfie (#6) super-resolved with scale-factors 2x, 3x, 4x, and 5x, respectively.

The test images are magnified by a scale-factor *s* and its performance evaluation is carried out. For visual comparison, test image (#6) is upscaled by a factor of 2x, 3x, 4x, and 5x by the proposed algorithm and is depicted in Figure 9. The ROI considered for visual evaluation is the texture of the shirt. It is observed from Figure 9 that the texture details are well preserved for 2x magnification. For 3x magnification, the proposed algorithm is able to preserve the fine texture details as the interleaved pattern in the shirt is clear to visualize. For 4x magnification, though the pattern in the ROI is visible, the fine details in it are lost. It is also observed that ringing artifacts along the edges affect the quality of the image. Furthermore, the texture details are lost for a magnification factor of 5x. The results are quantified by its PSNR values tabulated in Table 5.

### 4.5. Influence of Training Dataset

**Figure 10:**
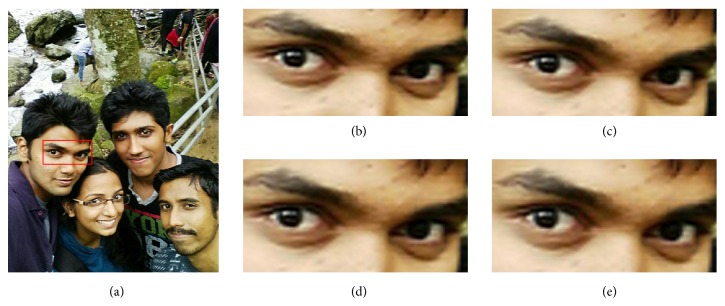
Visual comparison on test selfie (#2) for various training datasets (TR): (a) test selfie (#2), (b–e) magnified ROI of test selfie (#2) super-resolved with training datasets TR1, TR2, TR3, and TR4, respectively.

Training images captured by the rear camera of the smartphone can serve as fine exemplar to train the MVR operator. The performance of the proposed algorithm can be influenced by the training dataset used to train the MVR operator. To validate this, a performance evaluation based on variation in dataset is carried out. It is observed that the training images from the same device as the test image lead to better results than when the training and testing images are taken from different devices. For visual comparison, test selfie (#2) is super-resolved by a factor of 3x using the MVR operator trained by four different datasets and is depicted in Figure 10. The training dataset TR1 has a collection of random natural images as example images. Similarly, training datasets TR2, TR3, and TR4 have collection of example images captured by the rear camera of iPhone 4s, iPhone 6, and Nexus 5, respectively. From Figure 10(e), it is observed that the freckles beneath the eye are sharp and crisp for the image super-resolved using the MVR operator trained with TR4. This is due to the fact that both training examples in TR4 and the test selfie (#2) are captured by the same smartphone. As the rear camera of the smartphone is used by the same user, example images captured from the rear camera tend to possess similar low-level image features such as texture, granularity, and exposure as in the selfie image captured by front camera. In addition to this, the facial information contained in the selfies can possibly reoccur in the training set as it is captured by the same user. This self-similarity improves the interdependency between the images and results in a more robust and efficient MVR operator. The results are quantified by the PSNR values tabulated in Table 6.

## 5. Conclusion

In this paper, a fast example-based SR algorithm for super-resolving LR selfie image is presented. The proposed SR algorithm learns an optimal matrix-value regression (MVR) operator from a set of training samples captured from the rear camera of a smartphone. The relation between LR-HR training patch-pairs is established by an optimal MVR operator. It preserves structural similarity across training patch-pairs and effectively represents the image-level information of the training image patch-pairs. It is used effectively to super-resolve clean LR selfie image captured by the front camera of the smartphone and it is observed that the fine details in the super-resolved test selfie are preserved. In the future, the proposed algorithm will be extended to super-resolve distorted selfie images. Qualitative and quantitative experiments have validated the efficiency and effectiveness of the proposed algorithm over other state-of-the-art SR algorithms.

## Figures and Tables

**Algorithm 1:**
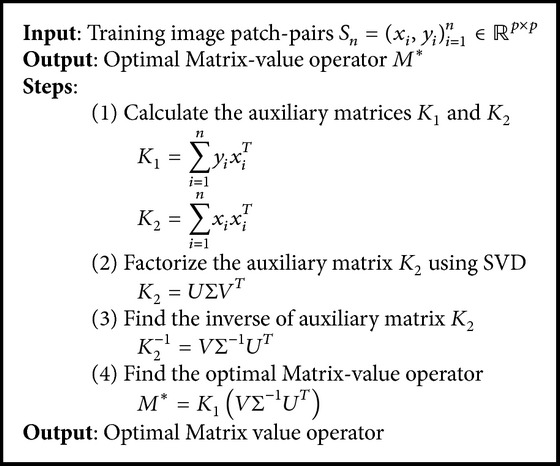
Algorithm to learn MVR operator.

**Algorithm 2:**
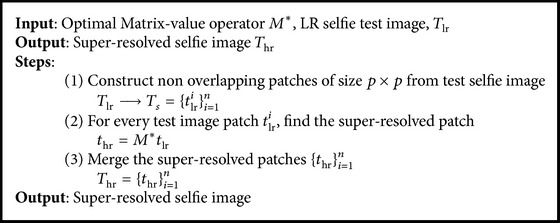
Algorithm for SR reconstruction.

**Table 1:** Ratio of rank deficient patches.

Patch-size/test image	Image 1	Image 2	Image 3	Image 4	Image 5	Average
3 × 3	0.6314	0.4290	0.6578	0.5808	0.4968	** 0.5591**
5 × 5	0.6935	0.5177	0.7276	0.6520	0.5649	** 0.6311**
7 × 7	0.6850	0.4866	0.7217	0.6305	0.5439	** 0.6135**
9 × 9	0.6423	0.4334	0.6779	0.5917	0.4741	** 0.5638**
11 × 11	0.5690	0.3268	0.6429	0.5336	0.4039	** 0.4952**

**Table 2:** Comparison of PSNR/SSIM with state-of-the-art SR algorithms for different test images with 3x magnification.

Test image	Yang et al.	Kim et al.	Dong et al.	He et al.	The proposed
Barbara	26.00/0.8847	26.36/0.8934	24.40/0.8341	25.72/0.8711	** 27.47/0.9430**
Lena	30.69/0.9421	32.67/0.9514	30.13/0.9146	31.28/0.9122	** 33.27/0.9763**
Selfie (#1)	29.21/0.9935	30.08/0.9962	27.92/0.9784	28.51/0.9917	** 31.69/0.9960**
Selfie (#2)	28.20/0.9885	28.80/0.9922	26.87/0.9558	27.39/0.9825	** 30.78/0.9928**
Selfie (#3)	26.01/0.9365	26.96/0.9486	24.39/0.8975	25.34/0.9226	** 27.87/0.9727**
Selfie (#4)	28.55/0.9241	29.19/0.9309	26.17/0.8682	28.58/0.9148	** 30.03/0.9603**
Selfie (#5)	31.45/0.9132	32.28/0.9309	29.87/0.8934	30.21/0.9021	** 32.02/0.9402**

**Table 3:** Comparison of computational efficiency of the proposed algorithm with various SR algorithms.

Algorithm	Training time	Recovery time (in seconds)			
		Lena	Test image (#1)	Test image (#3)	Test image (#5)
Dong et al.	—	352.6	468.4	186.2	261.4
He et al.	—	3360.3	3960.2	2460.8	3123.4
Yang et al.	>12 hours	11.8	24.1	18.2	29.6
Kim et al.	≈24 hours	32.6	41.8	26.3	38.1
Proposed	49 s	** 4.2**	** 2.6**	** 1.8**	** 2.8**

**Table 4:** Quantitative comparison on test selfie (#7) for various patch-sizes.

Patch-size	3 × 3	11 × 11	19 × 19	27 × 27	35 × 35	43 × 43

PSNR	31.02	31.13	31.22	31.43	31.32	31.31

**Table 5:** Quantitative comparison on test selfie (#6) for various scale-factors *s*.

Scale-factor	*s* = 2	*s* = 3	*s* = 4	*s* = 5

PSNR	28.92	27.66	27.31	24.80

**Table 6:** Quantitative comparison on test selfie (#2) for various training datasets.

Training dataset	TR1	TR2	TR3	TR4
PSNR	30.48	30.57	30.55	** 30.78 **
